# Flexibility evaluation of multiechelon supply chains

**DOI:** 10.1371/journal.pone.0194050

**Published:** 2018-03-27

**Authors:** João Flávio de Freitas Almeida, Samuel Vieira Conceição, Luiz Ricardo Pinto, Ricardo Saraiva de Camargo, Gilberto de Miranda Júnior

**Affiliations:** 1 Dept. of Industrial Engineering, Federal University of Minas Gerais, Belo Horizonte, Minas Gerais, Brazil; 2 Dept. of Applied Mathematics, Federal University of Espírito Santo, São Mateus, Espírito Santo, Brazil; Southwest University, CHINA

## Abstract

Multiechelon supply chains are complex logistics systems that require flexibility and coordination at a tactical level to cope with environmental uncertainties in an efficient and effective manner. To cope with these challenges, mathematical programming models are developed to evaluate supply chain flexibility. However, under uncertainty, supply chain models become complex and the scope of flexibility analysis is generally reduced. This paper presents a unified approach that can evaluate the flexibility of a four-echelon supply chain via a robust stochastic programming model. The model simultaneously considers the plans of multiple business divisions such as marketing, logistics, manufacturing, and procurement, whose goals are often conflicting. A numerical example with deterministic parameters is presented to introduce the analysis, and then, the model stochastic parameters are considered to evaluate flexibility. The results of the analysis on supply, manufacturing, and distribution flexibility are presented. Tradeoff analysis of demand variability and service levels is also carried out. The proposed approach facilitates the adoption of different management styles, thus improving supply chain resilience. The model can be extended to contexts pertaining to supply chain disruptions; for example, the model can be used to explore operation strategies when subtle events disrupt supply, manufacturing, or distribution.

## Introduction

Supply chains (SCs) are required to handle environmental uncertainties. Therefore, it is important to develop strategies to improve the flexibility and resilience of SCs without compromising their operation efficiency and effectiveness [1]. In a generic system, suppliers transport raw materials in multiple lots to factories. Manufacturing processes convert these materials into finished products. Periodically, these products need to be transported to other factories, to distribution centers, or directly to customers. SCs that decide to maintain a high level of finished goods inventories combined with fast shipping fleets increase their chances of meeting customer demand on time, thus improving their service level and revenue. However, SCs also need to maintain low inventory levels and choose economic modes of transport to minimize production, inventory, and logistics costs. The allocation of production capacity, inventories, and transportation simultaneously to satisfy demands can be very challenging. Nevertheless, to develop a tactical SC plan, these interactions must be considered.

In uncertain environments, an important requirement for SC planning models is the ability to cope with flexibility. However, in many situations, the parameters of deterministic models are not known completely. In such cases, sensitivity analysis combined with parametric optimization is commonly adopted. However, this strategy, known as parametric linear programming, is hardly relevant to optimization under uncertainty. This strategy makes predictions only when a model faces certainty scenarios [2], [3]. Therefore, during optimization, the proper approach to handle uncertainty is to use stochastic models. However, when the number of scenarios increases, stochastic SC planning models become complex and difficult to solve. Flexibility analysis often considers only one or two echelons of SCs.

The present study is aimed at investigating a four-echelon SC tactical planning model by robust stochastic programming. To the best of our knowledge, the adopted approach has not yet been explored in previous studies. This study intends to bridge this gap. The proposed model combines robust optimization and stochastic programming features to evaluate SC flexibility and resilience, considering scenarios with stochastic parameters and adjustable levels of demand variability.

The rest of the paper is organized as follows: In the next section, the relevant literature on SC planning under uncertainty is revisited. Then, the SC problem is presented in terms of a numerical example with deterministic parameters, and the proposed formulation for the stochastic problem is explained. In the section following this, a computational study is presented. SC flexibility was evaluated in three dimensions: supply, production, and distribution. The model also enabled the analysis that considers the tradeoff between demand variability and the service level. Finally, the conclusions and directions for future research are presented.

## Literature review

The assumption that a system will operate in a stable environment is not realistic. Customers can change their needs, suppliers can offer discounts on products, and regulations can impact transportation costs. To achieve SC flexibility, uncertainty must be considered in systems and models [4]. The idea of incorporating uncertainty in mathematical programming models was pioneered by Dantzig [5]. Since then, the understanding of uncertainty via stochastic programming has progressed [6], [7]. Particularly, SC planning models have been reviewed by some researchers, e.g., Birge [8], Mula et al. [9], and Sodhi and Tang [10]. Uncertainty can also be modeled by robust optimization.

Through robust optimization, a set of computationally tractable uncertainty scenarios can be selected and evaluated simultaneously; this approach was initially proposed by Soyster [11] and Falk [12]. Recent advances in robust optimization have been presented by Gabriel et al. [13]. The approach is an alternative to the use of sensitivity analysis because the latter is a reactive post-optimality study that cannot assess the impact of data uncertainty. In robust optimization, the knowledge of probability distribution parameters is not previously assumed. Its use is motivated by the tradeoff between the value of the objective function and the risk of infeasibility in cases where sufficient reliable data are not available to elaborate decision scenarios [14], [15], [16]. It was developed to ensure problem feasibility given a set of realizations of uncertainty. However, the model can become very conservative, in which case, one can previously set the uncertainty budgets [17].

For SC planning under uncertainty, stochastic programming and robust optimization have been successfully adopted. Stochastic programming formulations are frequently used because they usually yield good expected performance estimates [18]. On the other hand, robust optimization can favor SC planning systems that need to handle parameter uncertainties [19], [20]. Some applications include reverse logistics [21] and manufacturing under the build-to-order strategy [22].

Recent studies on SC flexibility include: the use of decomposition approaches for solving large-scale models to improve SC resilience [23]; the study of cycle and delivery times for SC systems considering failure in rework [24]; multiobjective simulation-based optimization to evaluate supplier flexibility and safety stock levels [25]; the development of closed-loop SC models for evaluating uncertainty on demand and returns for tire remanufacturing [26], and the evaluation of corporate social responsibility [27].

A recent review on SC flexibility identified the use of quantitative models as a new research direction for achieving tradeoff between performance measures [28]. To the best of our knowledge, no work has evaluated four-echelon SC flexibility by simultaneously considering demand variability and service levels for supply, production, and distribution scenarios. To address this challenge, we develop a hybrid model that combines stochastic programming and robust optimization for tactical policies. The model also includes a bill of materials for a generic product structure and one sublevel. The supply–production–distribution model is capacitated, multiplant, multiproduct, multiperiod, and multimodal. Table 1 presents the recent and relevant works that contribute to the proposed integrated approach of evaluating the flexibility of the tactical supply chain planning problem using two-stage stochastic programming and robust optimization.

**Table 1:** Recent and relevant works that contribute to the proposed approach for supply chain planning.

Author(s)	Year	Tactical level	Uncertainty	2SSP^1^	RO^2^	4ESC^3^ Flexibility
[29]	2000			✓		
[18]	2003		✓	✓		
[16]	2004	✓	✓	✓		
[20]	2005	✓	✓		✓	
[9]	2006	✓	✓	✓		
[10]	2009	✓	✓			
[21]	2011				✓	✓
[22]	2013	✓	✓		✓	
[17]	2013		✓	✓	✓	
[13]	2014				✓	
[23]	2014			✓		✓
[1]	2015		✓			✓
[28]	2016	✓	✓			✓
[24]	2016	✓	✓			
[26]	2017		✓			✓
[25]	2017	✓	✓			

^1^Two-Stage Stochastic Programming.

^2^Robust Optimization.

^3^Four-Echelon SC.

## Problem definition and modeling

The proposed multiechelon supply chain model considers a two-stage stochastic programming formulation [6] with robust optimization elements for the objective function and constraints [14]. For readability we consider the following nomenclature: MESC-2SSP-RO. Probability distributions represent the price ranges and demand values for uncertainty modeling. The goal is to find an optimal policy that maximizes the expected profit. The stochastic model is risk neutral and insensitive to results that are far from the expected solution. The objective function assumes the generic nonlinear form according to Eq (1):

$$\begin{array}{c} \max\;c^{⊤}x+E_{\omega \in \Omega }\left[Q\left(x,\omega \right)\right]-\alpha f\left(\omega ,y\right) \end{array}$$ (1)

where *f* is the variance of second-stage costs and *α* is a scalar used by the decision maker to determine nonnegative risk tolerance. Larger values of *α* produce solutions that reduce the variance, whereas smaller values of *α* increase the expected profit. The exploitation of this formulation gives quadratic terms. We linearize the mean absolute deviation of the objective function [19]. The new formulation on Eq (2) and constraints (3) and (4) reduces the computational effort by using half the variables used in the generic Eq (1):

$$\begin{array}{c} \max\Psi =\sum_{s\in X}\rho_{s}\xi_{s}-\lambda \sum_{s\in X}\rho_{s}\left[\left(\xi_{s}-\sum_{s^{\prime }\in X}\rho_{s^{\prime }}\xi_{s^{\prime }}\right)+2\theta_{s}\right]-\omega \sum_{s\in X}\rho_{s}\delta_{s} \end{array}$$ (2)

$$\begin{array}{c} \left(\sum_{s^{\prime }\in X}\rho_{s^{\prime }}\xi_{s^{\prime }}-\xi_{s}\right)\leq \theta_{s}\;\forall s\in S \end{array}$$ (3)

$$\begin{array}{c} \theta_{s}\geq 0\;\forall s\in S \end{array}$$ (4)

where *θ*_*s*_ represents the average deviation violation of scenario *s* from the *X* scenarios. λ is a weight that measures the tradeoff between risk and the expected value. In Eq (2) and constraints (3), if *ξ*_*s*_ − ∑_*s*′∈*X*_ *ρ*_*s*′_ *ξ*_*s*′_ ≥ 0, then *θ*_*s*_ = 0 under the optimal plan and Ψ = ∑_*s*∈*X*_ *ρ*_*s*_ *ξ*_*s*_ + λ∑_*s*∈*X*_ *ρ*_*s*_(*ξ*_*s*_ − ∑_*s*′∈*X*_ *ρ*_*s*′_ *ξ*_*s*′_). Else, if *ξ*_*s*_ − ∑_*s*′∈*X*_ *ρ*_*s*′_ *ξ*_*s*′_ ≤ 0, then *θ*_*s*_ = ∑_*s*′∈*X*_ *ρ*_*s*′_ *ξ*_*s*′_ − *ξ*_*s*_ under the optimal plan and Ψ = ∑_*s*∈*X*_ *ρ*_*s*_ *ξ*_*s*_ + λ∑_*s*∈*X*_ *ρ*_*s*_(∑_*s*′∈*X*_ *ρ*_*s*′_ *ξ*_*s*′_ − *ξ*_*s*_). The second and third terms of Eq (2) refer to the solution and model robustness, respectively. When solution robustness is ensured, the solution is close to optimality under any scenario, whereas when model robustness is ensured, the solution satisfies the demand under any scenario. *δ*_*s*_ represents a negative deviation from the demand, that is, a situation in which customers face a lack of products, whereas *ω* represents a weight of penalty over *δ*_*s*_ for the tradeoff between model robustness and solution robustness.

### Notation and model formulation

**Fig 1:**
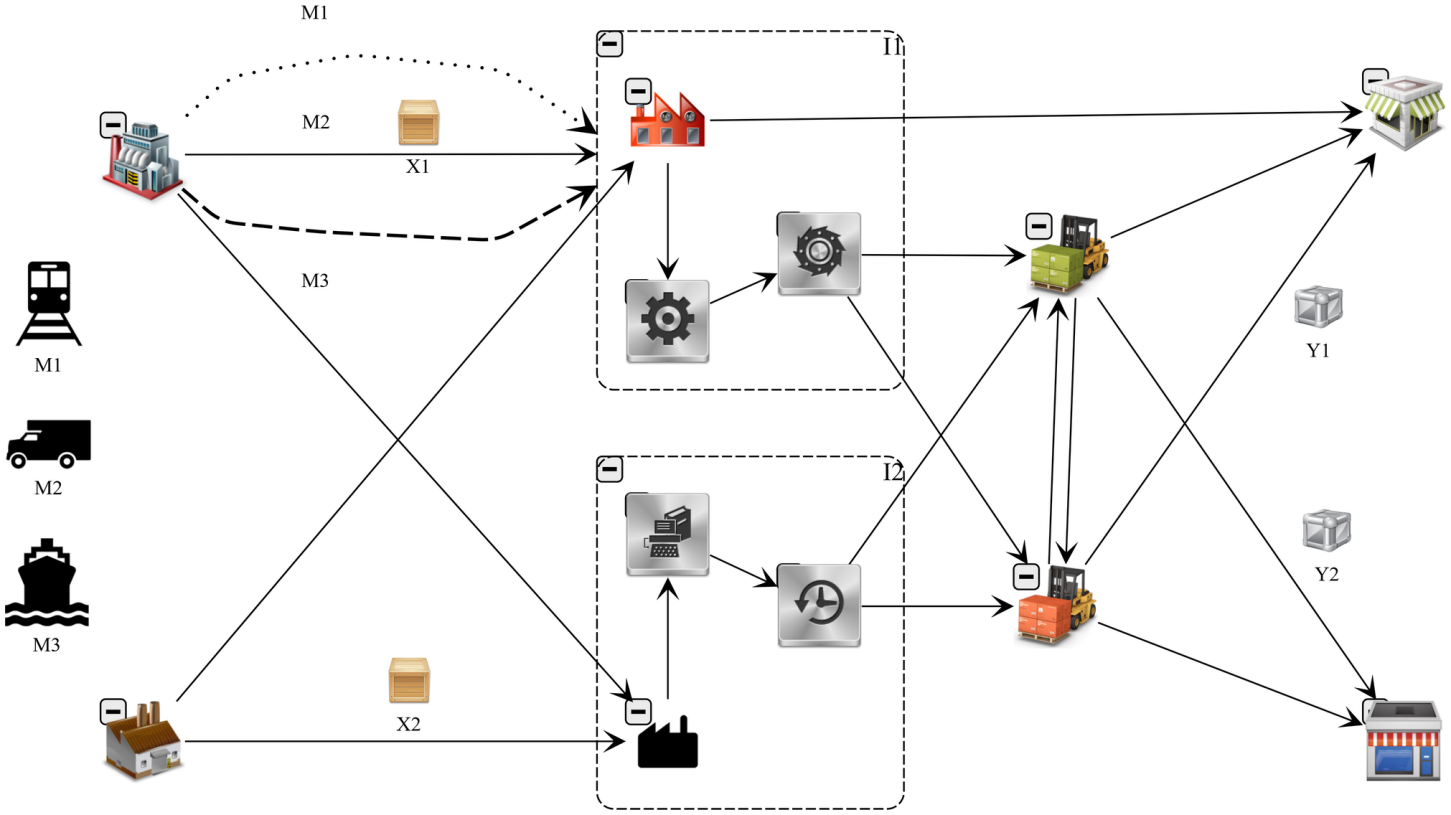
Four-echelon SC. The supply–production–distribution model is capacitated, multiplant, multiproduct, multiperiod, and multimodal.

Consider a SC where P is the set of products consisting of X raw materials and Y finished products (i.e. P=X∪Y). Let L be a location in a network consisting of F suppliers, I industrial plants, H distribution hubs, and C customers; thus, L=F∪I∪H∪C. In this four-echelon SC, F suppliers provide X raw materials to I industrial plants. These plants process raw materials on R resources, thus producing Y finished products over T periods to meet the demands of C customers. The sets of the products, locations, resources, and periods are indexed by *p*, *l*, *r* and *t*, respectively. Fig 1 shows a schematic of the SC.

We list the deterministic and stochastic parameters in Table 2 and the first and second-stage variables in Table 3. The elements of the objective function (13) are described in Table 4.

**Table 2:** MESC-2SSP-RO model parameters.

**Deterministic parameters**	
$T_{lrp}^{R}$	∈{0, 1}: Technical route of product *p* on resource *r* at location *l*
*B*_*p*′*p*_	Bill of materials *p*′ required to produce a unit of product *p*
$M_{lrp}^{C}$	Unit time required to produce product *p* on resource *r* at location *l*
$E_{lrt}^{F}$	Resource efficiency *r* at location *l* in period *t*
$A_{t}^{H}$	Available hours in each period *t*
$A_{lrt}^{X}$	Extra hours available on resource *r* at location *l* in period *t*
$L_{lp}^{M}$	Lot size of product *p* at location *l*
$P_{lrt}^{M}$	Hours of preventive maintenance required for resource *r* at location *l* in period *t*
$S_{lpt}^{S}$	Safety stock of product *p* at location *l* in period *t*
$S_{lpt}^{X}$	Stock capacity of product *p* at location *l* in period *t*
$A_{lpt}^{R}$	Availability of raw materials for product *p* at location *l* in period *t*
$S_{lp}^{0}$	Initial inventory of product *p* at location *l*
*Y*_*lr*_	Raw material yield on resource *r* at location *l*
$N_{lr}^{M}$	Number of resources of type *r* at location *l*
$T_{mll^{\prime }}^{CX}$	Transportation capacity of raw-material on modal *m* from location *l* to *l*′
$T_{mll^{\prime }}^{CY}$	Transportation capacity of finished product on modal *m* from location *l* to *l*′
$C_{ht}^{I}$	Inbound handling capacity at location *l* in period *t*
$C_{ht}^{O}$	Outbound handling capacity on location *l* and time period *t*
$A_{lrt}^{V}$	$\left(A_{t}^{H}N_{lr}^{M}-P_{lrt}^{M}\right)\left(E_{lrt}^{F}Y_{lr}\right)$ Available hours on resource *r* at location *l* in period *t*
**Stochastic parameters**	
*D*_*cpts*_	Demand of customer *c* for product *p* in period *t* and scenario *s*
*R*_*ps*_	Sales revenue of finished product *p* in scenario *s*
*N*_*ps*_	Fictitious cost penalty for not meeting demand *p* in scenario *s*
$C_{lrs}^{F}$	Fixed cost of resource *r* at location *l* in scenario *s*
$C_{lps}^{V}$	Variable cost of production of *p* at location *l* in scenario *s*
$C_{lrs}^{X}$	Extra capacity cost on resource *r* at location *l* in scenario *s*
$C_{lps}^{S}$	Unit inventory cost of product *p* at location *l* in scenario *s*
$C_{mll^{\prime }s}^{L}$	Unit transport cost on modal *m* from location *l* to *l*′ in scenario *s*
$C_{lps}^{P}$	Unit procurement cost of raw material *x* at location *l* in scenario *s*
$T_{lps}^{X}$	Tax over finished product *y* sold to customer *c* in scenario *s*
*ρ*_*s*_	Probability of each scenario *s*, $\sum_{s\in S}\rho_{s}=1$
λ	Weight for measuring tradeoff between risk and expected value
*ω*	Penalty for measuring tradeoff between solution and model robustness

**Table 3:** MESC-2SSP-RO model decision variables.

**First-stage decision variables**	
*α*_*lp*_	$\in Z^{+}$: Production of product *p* at location *l* in first period
*a*_*lrp*_	Production of product *p* on resource *r* at location *l* in first period
*b*_*lp*_	Consumption of raw material *x* at location *l* in first period
*s*_*lpt*_	Stock of product *p* at location *l* at the end of first period
*d*_*lp*_	Met demand of product *p* at location *l* in first period
*n*_*lp*1*s*_	Nonsatisfied demand of product *p* at location *l* in first period in scenario *s*
*r*_*lp*_	$\in Z^{+}$: Procurement of lots of raw material *x* at location *l* in first period
*t*_*mll*′*p*_	Quantity of product *p* transported on modal *m* from *l* to *l*′
*c*_*lr*_	Consumption of resource *r* at location *l* in first period
$c_{lr}^{\prime }$	Overtime percentage for resource *r* at location *l*
*y*_*lr*_	∈ {0, 1}: Decision of activate (or not activate) resource *r* at location *l* in first period
**Second-stage decision variables**	
*α*_*lpts*_	$\in Z^{+}$: Production of product *p* at location *l* in period *t* and scenario *s*
*a*_*lrpts*_	Production of product *p* on resource *r* at location *l* in period *t* and scenario *s*
*b*_*lpts*_	Consumption of raw material *x* at location *l* in period *t* and scenario *s*
*s*_*lpts*_	Stock of product *p* at location *l* at end of period *t* in scenario *s*
*d*_*lpts*_	Met demand of product *p* at location *l* in period *t* and scenario *s*
*n*_*lpts*_	Nonsatisfied demand of product *p* at location *l* in period *t* and scenario *s*
*r*_*lpts*_	$\in Z^{+}$: Procurement of lots of raw material *x* at location *l* in period *t* and scenario *s*
*t*_*mll*′*pts*_	Quantity of product *p* transported on modal *m* from *l* to *l*′ in period *t* and scenario *s*
*c*_*lrts*_	Consumption of resource *r* at location *l* in period *t* and scenario *s*
$c_{lrts}^{\prime }$	Overtime percentage for resource *r* at location *l* in period *t* and scenario *s*
*y*_*lrts*_	∈ {0, 1}: Decision to activate (or not activate) resource *r* at location *l* in period *t* and scenario *s*
*θ*_1__*s*_	Deviation of mean violation in first-stage scenario *s*
*θ*_2__*s*_	Deviation of mean violation in second-stage scenario *s*

**Table 4:** Objective function elements of four-echelon SC planning model.

Elements^a^	Description
$R_{1}^{P}$	Revenue after taxes from sales in first stage
$C_{1}^{L}$	Logistics cost from origin to destination on different transport modes in first stage
$C_{1}^{F}$	Fixed cost for machine activation in each plant in first stage
$C_{1}^{V}$	Finished product cost in each plant in first stage
$C_{1}^{P}$	Procurement cost of raw material in first stage
$C_{1}^{S}$	Storage cost in each plant in first stage
$C_{1}^{X}$	Capacity expansion cost of resources in each plant in first stage
$C_{1}^{N}$	Nondelivery cost for each customer in first stage
$R_{2}^{P}$	Revenue after taxes from sales in second stage
$C_{2}^{L}$	Logistics cost from origin to destination on different transport modes in second stage
$C_{2}^{F}$	Fixed cost for machine activation in each plant in second stage
$C_{2}^{V}$	Finished product cost in each plant in second stage
$C_{2}^{P}$	Procurement cost of raw material in second stage
$C_{2}^{S}$	Storage cost in each plant in second stage
$C_{2}^{X}$	Capacity expansion cost of resources in each plant in second stage
$C_{2}^{N}$	Nondelivery cost for each customer in second stage

^a^Elements subscript 1 and 2 represents first stage_[1]_ and second stage_[2]_.

Where: $R_{1}^{P}=\sum_{l\in C}\sum_{p\in Y}\left(R_{ps}-T_{lps}^{X}\right)d_{lp}$, $C_{1}^{L}=\sum_{m\in M}\sum_{ll^{\prime }\in K}\sum_{p\in Y}C_{mll^{\prime }s}^{L}t_{mll^{\prime }p}$, $C_{1}^{F}=\sum_{l\in I}\sum_{r\in R}C_{lrs}^{F}y_{lr}$, $C_{1}^{V}=\sum_{l\in I}\sum_{p\in Y}C_{lps}^{V}\alpha_{lp}$, $C_{1}^{P}=\sum_{l\in F}\sum_{p\in P}C_{lps}^{P}r_{lp}$, $C_{1}^{S}=\sum_{l\in I}\sum_{p\in Y}C_{lps}^{S}s_{lp}$, $C_{1}^{X}=\sum_{l\in I}\sum_{r\in R}C_{lrs}^{X}c_{lr}^{\prime }$, $C_{1}^{N}=\sum_{s\in S}\sum_{l\in C}\sum_{p\in Y}N_{ps}n_{lp}$, $R_{2}^{P}=\sum_{l\in C}\sum_{p\in Y}\sum_{t>1}\left(R_{ps}-T_{lps}^{X}\right)d_{lpts}$, $C_{2}^{F}=\sum_{l\in I}\sum_{r\in R}\sum_{t>1}C_{lrs}^{F}y_{lrts}$, $C_{2}^{L}=\sum_{m\in M}\sum_{ll^{\prime }\in K}\sum_{p\in Y}\sum_{t>1}C_{mll^{\prime }s}^{L}t_{mll^{\prime }pts}$, $C_{2}^{V}=\sum_{l\in I}\sum_{p\in Y}\sum_{t>1}C_{lps}^{V}\alpha_{lpts}$, $C_{2}^{P}=\sum_{l\in F}\sum_{p\in P}\sum_{t>1}C_{lps}^{P}r_{lpts}$, $C_{2}^{S}=\sum_{l\in I}\sum_{p\in Y}\sum_{t>1}C_{lps}^{S}s_{lpts}$, $C_{2}^{X}=\sum_{l\in I}\sum_{r\in R}\sum_{t>1}C_{lrs}^{X}c_{lrts}^{\prime }$ and $C_{2}^{N}=\sum_{s\in S}\sum_{l\in C}\sum_{p\in Y}N_{ps}n_{lpts}$.

The robust stochastic programming model is described as follows:

$$\begin{array}{c} \max\Psi =\sum_{s\in S}\rho_{s}\left(R_{1}^{P}-C_{1}^{L}-C_{1}^{F}-C_{1}^{V}-C_{1}^{P}-C_{1}^{S}-C_{1}^{X}\right)- \end{array}$$ (5)

$$\begin{array}{c} \lambda \sum_{s\in S}\rho_{s}[\left(R_{1}^{P}-C_{1}^{L}-C_{1}^{F}-C_{1}^{V}-C_{1}^{P}-C_{1}^{S}-C_{1}^{X}\right) \end{array}$$ (6)

$$\begin{array}{c} -\sum_{s^{\prime }\in S}\rho_{s^{\prime }}\left(R_{1^{\prime }}^{P}-C_{1^{\prime }}^{L}-C_{1^{\prime }}^{F}-C_{1^{\prime }}^{V}-C_{1^{\prime }}^{P}-C_{1^{\prime }}^{S}-C_{1^{\prime }}^{X}\right)+2{\theta_{1}}_{s}]-\omega C_{1}^{N}\rho_{s}+ \end{array}$$ (7)

$$\begin{array}{c} \sum_{s\in S}\rho_{s}\left(R_{2}^{P}-C_{2}^{L}-C_{2}^{F}-C_{2}^{V}-C_{2}^{P}-C_{2}^{S}-C_{2}^{X}\right)- \end{array}$$ (8)

$$\begin{array}{c} \lambda \sum_{s\in S}\rho_{s}[\left(R_{2}^{P}-C_{2}^{L}-C_{2}^{F}-C_{2}^{V}-C_{2}^{P}-C_{2}^{S}-C_{2}^{X}\right) \end{array}$$ (9)

$$\begin{array}{c} -\sum_{s^{\prime }\in S}\rho_{s^{\prime }}\left(R_{2^{\prime }}^{P}-C_{2^{\prime }}^{L}-C_{2^{\prime }}^{F}-C_{2^{\prime }}^{V}-C_{2^{\prime }}^{P}-C_{2^{\prime }}^{S}-C_{2^{\prime }}^{X}\right)+2{\theta_{2}}_{s}]-\omega C_{2}^{N}\rho_{s} \end{array}$$ (10)

Objective function of MESC-2SSP-RO on Eqs (5)–(10) maximizes the expected profit. It follows the robust two-stage stochastic programming formulation presented in Eq (2), where *ρ*_*s*_ is the occurrence probability of each scenario *s*; $\sum_{s\in S}\rho_{s}=1$, λ is a weight used to measure the tradeoff between risk and the expected value; and *ω* is a penalty used to measure the tradeoff between the solution and model robustness. The objective function is obtained as the difference between the after-tax revenue and the costs of procurement, production, inventory, and transportation. The costs consist of fixed and variable parts. Fixed costs are incurred on resource activation during certain periods. Variable costs are incurred on different levels of procurement, production, extra capacity use, inventory, logistics, and delivery.

$$\begin{array}{c} s_{lpt}=S_{lp}^{0}\;\;\;\;\;\;\;\forall l\in \left(I\cup H\right),p\in P,t=0 \end{array}$$ (11)

$$\begin{array}{c} S_{lpt}^{S}\leq s_{lpt}\leq S_{lpt}^{X}\;\;\;\;\;\;\forall l\in \left(I\cup H\right),p\in P,t=1 \end{array}$$ (12)

$$\begin{array}{c} S_{lpt}^{S}\leq s_{lpts}\leq S_{lpt}^{X}\;\;\;\;\forall l\in \left(I\cup H\right),p\in P,t\in 2..\left|T\right|,s\in S \end{array}$$ (13)

$$\begin{array}{c} L_{lp}^{M}r_{lp}\leq A_{lpt}^{R}\;\;\;\;\;\;\;\;\forall l\in F,p\in P,t=1 \end{array}$$ (14)

$$\begin{array}{c} L_{lp}^{M}r_{lpts}\leq A_{lpt}^{R}\;\;\;\;\;\;\forall l\in F,p\in P,t\in 2..\left|T\right|,s\in S \end{array}$$ (15)

The objective function of MESC-2SSP-RO is subjected to constraints in the first (*t* = 1) and second (t=2..|T|) stages. Constraint (11) expresses the initial stocks of raw materials and finished products that are considered to exist in industrial plants and distribution centers. Constraints (12) and (13) describe the stored volumes of raw materials and finished products that must consider the inventory safety levels and must not exceed the storage capacity limits of these locations. Constraints (14) and (15) indicate that the purchase of lots of raw materials or finished products must consider their availability with suppliers in each period.

$$\begin{array}{c} L_{lp}^{M}r_{lp}=\sum_{m\in M}\sum_{ll^{\prime }\in K}t_{mll^{\prime }p}\;\;\;\;\;\;\forall l\in F,p\in P,t=1 \end{array}$$ (16)

$$\begin{array}{c} L_{lp}^{M}r_{lpts}=\sum_{m\in M}\sum_{ll^{\prime }\in K}t_{mll^{\prime }pts}\;\;\;\forall l\in F,p\in P,t\in 2..\left|T\right|,s\in S \end{array}$$ (17)

$$\begin{array}{c} \sum_{m\in M}\sum_{l^{\prime }l\in K}t_{ml^{\prime }lp}+s_{lpt_{-1}}=s_{lp}+b_{lp}\;\;\;\forall l\in I,p\in X,t=1 \end{array}$$ (18)

$$\begin{array}{c} \sum_{m\in M}\sum_{l^{\prime }l\in K}t_{ml^{\prime }lpts}+s_{lpt_{-1}s}=s_{lpts}+b_{lpts}\forall l\in I,p\in X,t\in 2..\left|T\right|,s\in S \end{array}$$ (19)

$$\begin{array}{ccc} L_{lp}^{M}\alpha_{lp}+\sum_{m\in M}\sum_{l^{\prime }l\in K}t_{ml^{\prime }lp}+s_{lpt_{-1}} & = & \sum_{m\in M}\sum_{ll^{\prime }\in K}t_{mll^{\prime }p}+s_{lpt} \end{array}$$ (20)

$$\begin{array}{c} \forall l\in I,p\in Y,t=1 \end{array}$$ (21)

$$\begin{array}{ccc} L_{lp}^{M}\alpha_{lpts}+\sum_{m\in M}\sum_{l^{\prime }l\in K}t_{ml^{\prime }lpts}+s_{lpt_{-1}s} & = & \sum_{m\in M}\sum_{ll^{\prime }\in K}t_{mll^{\prime }pts}+s_{lpts} \end{array}$$ (22)

$$\begin{array}{c} \forall l\in I,p\in Y,t\in 2..|T|,S \end{array}$$ (23)

$$\begin{array}{ccc} \sum_{m\in M}\sum_{l^{\prime }l\in K}t_{ml^{\prime }lp}+s_{lpt_{-1}} & = & \sum_{m\in M}\sum_{ll^{\prime }\in K}t_{mll^{\prime }p}+s_{lpt}\;\forall l\in H,p\in Y,t=1 \end{array}$$ (24)

$$\begin{array}{ccc} \sum_{m\in M}\sum_{l^{\prime }l\in K}t_{ml^{\prime }lpts}+s_{lpt_{-1}s} & = & \sum_{m\in M}\sum_{ll^{\prime }\in K}t_{mll^{\prime }pts}+s_{lpts} \end{array}$$ (25)

$$\begin{array}{c} \forall l\in H,p\in Y,t\in 2..|T|,S \end{array}$$ (26)

$$\begin{array}{c} \sum_{m\in M}\sum_{l^{\prime }l\in K}t_{ml^{\prime }lp}=d_{lp}\;\;\;\;\;\;\forall l\in C,p\in Y,t=1 \end{array}$$ (27)

$$\begin{array}{c} \sum_{m\in M}\sum_{l^{\prime }l\in K}t_{ml^{\prime }lpts}=d_{lpts}\;\;\;\;\forall l\in C,p\in Y,t\in 2..\left|T\right|,s\in S \end{array}$$ (28)

Flow balance constraints (16)–(28) integrate the different parts of the problem. The end of each period is connected by the sum of the input and output flows; therefore, the transportation of products is not allowed if the product does not reach the destination within the planned horizon. The input and output flows are considered for each location, product, and period. The input flow is represented by the transportation of raw materials or finished products from the previous SC echelon, the production of finished products, the inventory level, and the procurement of raw materials or finished products at the end of the previous period. The output flow is the result of the balance of transportation of items to the next SC echelon, the met demand, the inventory level, and the consumption of raw materials in the production process at the end of period.

$$\begin{array}{c} \sum_{m\in M}\sum_{l^{\prime }l\in K}\sum_{p\in Y}t_{ml^{\prime }lp}\leq C_{lt}^{I}\;\;\;\;\;\;\forall l\in H,t=1 \end{array}$$ (29)

$$\begin{array}{c} \sum_{m\in M}\sum_{l^{\prime }l\in K}\sum_{p\in Y}t_{ml^{\prime }lpts}\leq C_{lt}^{I}\;\;\;\;\forall l\in H,t\in 2..\left|T\right|,s\in S \end{array}$$ (30)

$$\begin{array}{c} \sum_{m\in M}\sum_{ll^{\prime }\in K}\sum_{p\in Y}t_{mll^{\prime }p}\leq C_{lt}^{O}\;\;\;\;\;\;\forall l\in H,t=1 \end{array}$$ (31)

$$\begin{array}{c} \sum_{m\in M}\sum_{ll^{\prime }\in K}\sum_{p\in Y}t_{mll^{\prime }pts}\leq C_{lt}^{O}\;\;\;\;\forall l\in H,t\in 2..\left|T\right|,s\in S \end{array}$$ (32)

$$\begin{array}{c} \sum_{p\in Y}a_{lrp}M_{lrp}^{C}=c_{lr}\;\;\;\;\;\;\;\forall l\in I,r\in R,t=1 \end{array}$$ (33)

$$\begin{array}{c} \sum_{p\in Y}a_{lrpts}M_{lrp}^{C}=c_{lrts}\;\;\;\;\forall l\in I,r\in R,t\in 2..\left|T\right|,s\in S \end{array}$$ (34)

Constraints (29)–(32) represent the inbound and outbound handling capacities at the distribution centers for each period. The production in each process depends on the route and production time of each item. Eqs (33) and (34) describe the production capacity use. The maintenance of a process activated during low demand times may incur unnecessary fixed costs of operation. Temporary process stoppages are tactical decisions that can lead to a reduction in operation costs because teams would then be reallocated to alternate company sites for training and supporting activities.

$$\begin{array}{c} c_{lr}\leq AV_{lr}y_{lr}+c_{lr}^{\prime }A_{lrt}^{X}\;\;\;\;\;\;\forall l\in I,r\in R,t=1 \end{array}$$ (35)

$$\begin{array}{c} c_{lrts}\leq AV_{lrt}y_{lrts}+c_{lrt}^{\prime }A_{lrt}^{X}\;\;\;\forall l\in I,r\in R,t\in 2..\left|T\right|,s\in S \end{array}$$ (36)

Constraints (35) and (36) express the capacity of a process, which is measured by the total available production time. In this time period, a process may or may not be activated. If activated, its capacity can be reduced by performing scheduled preventive maintenance, controlling operational efficiency, or/and controlling raw material yield.

$$\begin{array}{c} c_{lp}^{\prime }\leq y_{lr}\;\;\;\;\;\;\;\;\;\forall l\in I,r\in R,t=1 \end{array}$$ (37)

$$\begin{array}{c} c_{lpts}^{\prime }\leq y_{lrts}\;\;\;\;\;\;\forall l\in I,r\in R,t\in 2..\left|T\right|,s\in S \end{array}$$ (38)

Constraints (37) and (38) indicate that the decision to use overtime can be a profitable alternative. The use of extra capacity results in extra costs, which are included in the objective function. However, the value of the extra costs is limited by the company. These constraints also guarantee that extra capacity can be activated only if there is a need for production in the period.

$$\begin{array}{c} a_{lrp}T_{lrp}^{R}=L_{lp}^{M}\alpha_{lp}\;\;\;\;\;\forall l\in I,r\in R,p\in P,t=1 \end{array}$$ (39)

$$\begin{array}{c} a_{lrpts}T_{lrp}^{R}=L_{lp}^{M}\alpha_{lpts}\;\;\;\forall l\in I,r\in R,p\in P,t\in 2..\left|T\right|,s\in S \end{array}$$ (40)

$$\begin{array}{c} b_{lp^{\prime }}=\sum_{p\in Y}B_{p^{\prime }p}L_{lp}^{M}\alpha_{lp}\;\;\;\;\;\;\forall l\in I,p^{\prime }\in X,t=1 \end{array}$$ (41)

$$\begin{array}{c} b_{lp^{\prime }ts}=\sum_{p\in Y}B_{p^{\prime }p}L_{lp}^{M}\alpha_{lpts}\;\;\;\forall l\in I,p^{\prime }\in X,t\in 2..\left|T\right|,s\in S \end{array}$$ (42)

$$\begin{array}{c} \sum_{p\in X}t_{mll^{\prime }p}\leq T_{mll^{\prime }}^{CX}\;\;\;\;\;\;\forall m\in M,ll^{\prime }\in K,t=1 \end{array}$$ (43)

$$\begin{array}{c} \sum_{p\in X}t_{mll^{\prime }pts}\leq T_{mll^{\prime }}^{CX}\;\;\;\;\forall m\in M,ll^{\prime }\in K,t\in 2..\left|T\right|,s\in S \end{array}$$ (44)

$$\begin{array}{c} \sum_{p\in Y}t_{mll^{\prime }p}\leq T_{mll^{\prime }}^{CY}\;\;\;\;\;\;\forall m\in M,ll^{\prime }\in K,t=1 \end{array}$$ (45)

$$\begin{array}{c} \sum_{p\in Y}t_{mll^{\prime }pts}\leq T_{mll^{\prime }}^{CY}\;\;\;\;\forall m\in M,ll^{\prime }\in K,t\in 2..\left|T\right|,s\in S \end{array}$$ (46)

$$\begin{array}{c} d_{lp}=D_{tpcs}-n_{lps}\;\;\;\;\;\;\forall l\in C,p\in Y,s\in S,t=1 \end{array}$$ (47)

$$\begin{array}{c} d_{lpts}=D_{tpcs}-n_{lpts}\;\;\;\;\;\forall l\in C,p\in Y,t\in 2..\left|T\right|,s\in S \end{array}$$ (48)

$$\begin{array}{c} b_{lp},s_{lpt},d_{lp},n_{lp}\geq 0\;\;\;\;\;\;\;\forall l\in L,p\in P,t=1 \end{array}$$ (49)

$$\begin{array}{c} b_{lpts},s_{lpts},d_{lpts},n_{lpts}\geq 0\;\;\;\forall l\in L,p\in P,t\in 2..\left|T\right|,s\in S \end{array}$$ (50)

$$\begin{array}{c} t_{mll^{\prime }p}\geq 0\;\;\;\;\;\;\;\;\forall m\in M,ll^{\prime }\in L,p\in P \end{array}$$ (51)

$$\begin{array}{c} t_{mll^{\prime }pts}\geq 0\;\;\;\;\forall m\in M,ll^{\prime }\in L,p\in P,t\in 2..\left|T\right|,s\in S \end{array}$$ (52)

$$\begin{array}{c} \alpha_{lp},r_{lp}\in Z^{+}\;\;\;\;\;\;\;\;\;\;\forall l\in L,p\in P \end{array}$$ (53)

$$\begin{array}{c} \alpha_{lpts},r_{lpts}\in Z^{+}\;\;\;\;\;\forall l\in L,p\in P,t\in 2..\left|T\right|,s\in S \end{array}$$ (54)

$$\begin{array}{c} a_{lrp}\geq 0\;\;\;\;\;\;\;\;\forall l\in I,r\in R,p\in P \end{array}$$ (55)

$$\begin{array}{c} a_{lrpts}\geq 0\;\;\;\;\;\forall l\in I,r\in R,p\in P,t\in 2..\left|T\right|,s\in S \end{array}$$ (56)

$$\begin{array}{c} y_{lr}\in \left\{0,1\right\}\;\;\;\;\;\;\;\;\;\forall l\in I,r\in R \end{array}$$ (57)

$$\begin{array}{c} y_{lrts}\in \left\{0,1\right\}\;\;\;\;\;\;\forall l\in I,r\in R,t\in 2..\left|T\right|,s\in S \end{array}$$ (58)

$$\begin{array}{c} 0\leq c_{lr}^{\prime }\leq 1\;\;\;\;\;\;\;\;\;\forall l\in I,r\in R \end{array}$$ (59)

$$\begin{array}{c} 0\leq c_{lrts}^{\prime }\leq 1\;\;\;\;\;\;\forall l\in I,r\in R,t\in 2..\left|T\right|,s\in S \end{array}$$ (60)

Constraints (39) and (40) ensure that a finished product is produced in the latest machine of the technical route in each manufacturing plant. Constraints (41) and (42) represent the bill of materials for a generic product structure [30]; therefore, a finished product is a result of the combination of raw materials in different proportions. Constraints (43)–(46) guarantee that the product flow does not exceed the transportation capacity for each transport mode. Constraints (47) and (48) indicate that eventually, some part of the original demand is not met. Constraints (49)–(60) characterize the domain of the variables.

$$\begin{array}{ccc} & & \sum_{s^{\prime }\in S}\rho_{s^{\prime }}\left(R_{1^{\prime }}^{P}-C_{1^{\prime }}^{L}-C_{1^{\prime }}^{F}-C_{1^{\prime }}^{V}-C_{1^{\prime }}^{P}-C_{1^{\prime }}^{S}-C_{1^{\prime }}^{X}\right)-\\ & & \;\left(R_{1}^{P}-C_{1}^{L}-C_{1}^{F}-C_{1}^{V}-C_{1}^{P}-C_{1}^{S}-C_{1}^{X}\right)\leq {\theta_{1}}_{s}\;\forall s\in S \end{array}$$ (61)

$$\begin{array}{ccc} & & \sum_{s^{\prime }\in S}\rho_{s^{\prime }}\left(R_{2^{\prime }}^{P}-C_{2^{\prime }}^{L}-C_{2^{\prime }}^{F}-C_{2^{\prime }}^{V}-C_{2^{\prime }}^{P}-C_{2^{\prime }}^{S}-C_{2^{\prime }}^{X}\right)-\\ & & \;\left(R_{2}^{P}-C_{2}^{L}-C_{2}^{F}-C_{2}^{V}-C_{2}^{P}-C_{2}^{S}-C_{2}^{X}\right)\leq {\theta_{2}}_{s}\;\forall s\in S \end{array}$$ (62)

$$\begin{array}{c} {\theta_{1}}_{s}\geq 0\;\;\;\;\;\;\;\;\;\;\;\forall s\in S \end{array}$$ (63)

$$\begin{array}{c} {\theta_{2}}_{s}\geq 0\;\;\;\;\;\;\;\;\;\;\;\forall s\in S \end{array}$$ (64)

As shown in constraint (3), the difference between the total average cost and the total cost of scenarios should be nonnegative and equivalent to the deviation for violating the average. These conditions are maintained by constraints (61)–(64). Constraints (61) and (63) determine these conditions for the first stage, whereas constraints (62) and (64) determine these conditions for the second stage of the robust two-stage stochastic program. These constraints ensure that distinct and nonnegative objective functions are generated by different scenarios.

### Numerical example

A numerical example is designed to demonstrate the scope of the proposed formulation. For didactic purposes, without loss of generality, we consider one scenario wherein parameters lambda and omega (penalty) equal zero, leading to a deterministic program. Parameters are kept simple so that the resulting plans, i.e., the purchasing, production, material use, storage, and transport levels, in each period can be intuitive. Although the model is simplified, it defines the optimal flow that maximizes the operating profit considering both production and logistics constraints throughout the four-echelon SC. The elements of this chain are listed in Table 5.

**Table 5:** Sets of numerical example.

Elements of numerical example	
Suppliers	*F*_1_, *F*_2_
Industrial Plants	*I*_1_, *I*_2_
Hubs of Distribution	*H*_1_, *H*_2_
Modal of Transport	*M*_1_, *M*_2_
Raw-materials	*X*_1_, *X*_2_
Finished products	*Y*_1_, *Y*_2_
Machines of Plant 1	*M*_*A*_, *M*_*B*_
Machines of Plant 2	*M*_*C*_, *M*_*D*_

**Fig 2:**
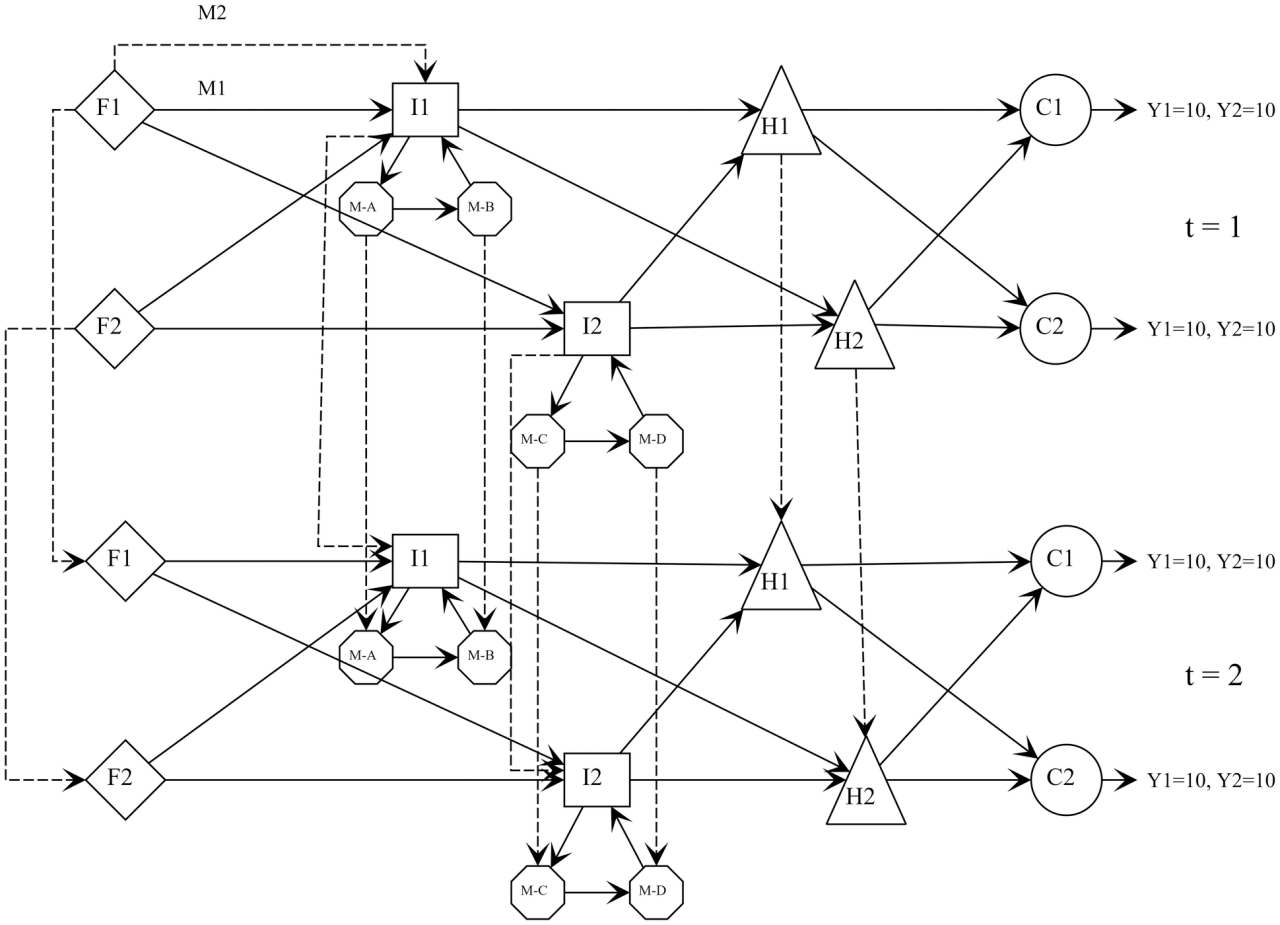
Schematic of numerical example SC. Supply, production, and distribution plan for two periods.

Fig 2 illustrates a small SC that plans its operations for the following two months. Two industrial plants sell two types of finished products to two customers. Each customer demands 10 units of each product in each month, i.e., *D*_*tpc*_ = 10, leading to a total demand of 80 units throughout the period. Products can be sent by two types of transport modes from industrial plants to two distribution centers and from there to the customers. The distribution hubs can handle the two products for dispatch or for stock, considering the safety stock, handling, and storage capacity limits.

**Fig 3:**
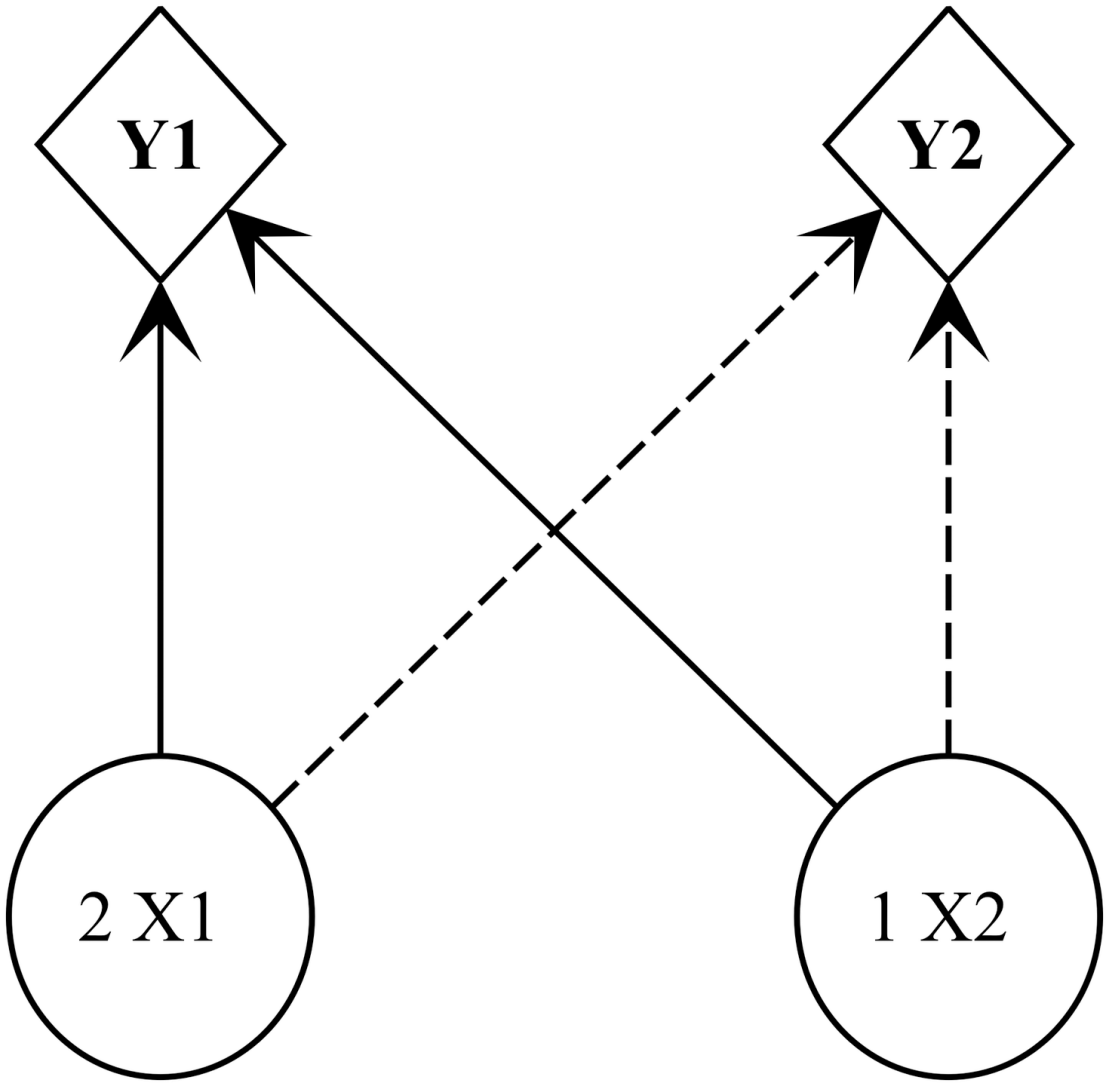
Generic product structure of the bill of materials. A generic product structure is adopted to set the raw materials used by finished products *Y*_1_ and *Y*_2_.

In the industrial plants, machines process the raw materials into finished products following a technology roadmap. Raw materials are processed by machines *M*_*A*_ and then *M*_*B*_ in plant *I*_1_ and by machines *M*_*C*_ and *M*_*D*_ in plant *I*_2_. The bill of materials considers a generic product structure and sets the raw materials used by each finished product, as illustrated in Fig 3. The industrial plants buy raw materials in multiple lots of 10 units from two suppliers. Finished products can be purchased only from supplier F1. Either of the two transport modes can be used to transport these items. Table 6 lists the availability of raw materials and finished products with suppliers.

**Table 6:** Monthly availability of raw materials and finished products.

Quantity	*X*_1_	*X*_2_	*Y*_1_	*Y*_2_
*F*_1_	50	10	10	10
*F*_2_	10	50	–	–

The initial stock in each plant is 100 units of each raw material and 5 units of each finished product. In both the distribution hubs, the initial stock is 5 units of each finished product. The safety stock of raw materials and finished products is 10 units for both the industrial plants and distribution hubs. Each machine has a capacity of 50 h and an extra capacity of 10 h, that is, $A_{irt}^{X}$ = 10. The production of finished product *Y*_1_ must occur in multiple lots of 5 units. There are no restrictions on the multiple lots for the production of finished product *Y*_2_. The transportation capacity per month is 10 units on transport mode *M*_1_ and 20 units on transport mode *M*_2_. The input and output material handling capacity is 50 units per month. Each finished product is sold at $100.00 to customers. A 5% tax is deducted from the sales revenue. The fixed cost of production for each machine is $500.00. Table 7 lists the variable production costs, whereas Table 8 lists the purchase costs of the raw materials or the finished product. The overtime cost for each machine is $100.00. The shipping cost using either of the transport modes is $10.00/(km⋅unit). For didactic purposes, without loss of generality, parameters $T_{lrp}^{R}$, $M_{lrp}^{C}$, $E_{irt}^{F}$, $N_{lr}^{M}$, *Y*_*lr*_ and $C_{lp}^{S}$ take the value of 1 for any set combination. No preventive maintenance is planned for the considered period, i.e., $P_{lrt}^{M}=0$.

**Table 7:** Production variable cost on each industrial plant.

Raw material/Product	*Y*_1_	*Y*_2_
*I*_1_	20	20
*I*_2_	10	20

**Table 8:** Procurement costs of raw material or finished products.

Procurement	*X*_1_	*X*_2_	*Y*_1_	*Y*_2_
*F*_1_	1	1	40	50
*F*_2_	1	1	–	–

The SC plan is presented in the following tables. The financial report and model statistics are given in Table 9. The entire demand is met, as listed in Table 10. From the gross sales revenue of $8,000.00, 5% tax is discounted, i.e., $400.00. In the production process, overtime is not necessary. In the optimal SC plan, production processes are partially activated. Table 11 indicates that production is complemented by the decision of buying finished products from a supplier.

**Table 9:** Finance performance and statistics of numerical example.

**Finance Report**	**Value ($)**
Gross revenues	8000.00
Net revenues	7600.00
Logistic cost	2000.00
Opportunity cost	0.00
Production fixed cost	2000.00
Production variable cost	940.00
Procurement cost	1002.00
Overtime cost	0.00
Inventory cost	80.00
Operating cost	1578.00
**Model Statistics**	**Value (unit.)**
Equations	687
Variables	606
Integer variables	128
Binary variables	8
Computational run time	0.1 s

**Table 10:** Demand meeting plan.

Month	Customer	Product	Quantity
1	*C*_1_	*Y*_1_	10
		*Y*_2_	10
	*C*_2_	*Y*_1_	10
		*Y*_2_	10
2	*C*_1_	*Y*_1_	10
		*Y*_2_	10
	*C*_2_	*Y*_1_	10
		*Y*_2_	10

**Table 11:** Procurement plan.

Month	Supplier	Item	Quantity
1	*F*_1_	*X*_1_	20
		*Y*_2_	10
2	*F*_1_	*Y*_2_	10

The purchasing plan determines the optimal flow of raw materials and finished products from suppliers to industrial plants considering the transportation capacity of each transport mode. Table 12 indicates that the optimal plan is to purchase 10 units of finished product *Y*_2_ in each month. However, this plan can be realized only with supplier *F*_1_, given the unavailability of these products with supplier *F*_2_. Furthermore, these products are transported only by transport mode *M*_2_ because the capacity of transport mode *M*_1_ is completely exhausted for the transportation of raw materials.

**Table 12:** Capacitated supply chain transportation plan.

Month	Modal	Origin	Destiny	Item	Quantity	Modal utilization
1	M1	*F*_1_	*I*_1_	*X*_1_	10	100%
			*I*_2_	*X*_1_	10	100%
		*I*_1_	*H*_1_	*Y*_2_	10	100%
			*H*_2_	*Y*_1_	5	50%
		*I*_2_	*H*_2_	*Y*_1_	10	100%
		*H*_1_	*C*_1_	*Y*_1_	10	100%
		*H*_2_	*C*_2_	*Y*_1_	10	100%
	M2	*F*_1_	*I*_1_	*Y*_2_	5	25%
			*I*_2_	*Y*_2_	5	25%
		*I*_1_	*H*_1_	*Y*_2_	15	75%
			*H*_2_	*Y*_2_	15	75%
		*I*_2_	*H*_1_	*Y*_1_	15	75%
			*H*_2_	*Y*_1_	20	100%
		*H*_1_	*C*_1_	*Y*_2_	10	50%
			*C*_2_	*Y*_2_	10	50%
2	M1	*I*_2_	*H*_1_	*Y*_2_	10	100%
		*H*_2_	*C*_1_	*Y*_1_	10	100%
			*C*_2_	*Y*_1_	10	100%
	M2	*F*_1_	*I*_2_	*Y*_2_	10	50%
		*H*_1_	*C*_1_	*Y*_2_	10	50%
		*H*_2_	*C*_2_	*Y*_2_	10	50%

Table 13 indicates that the inventories of the raw materials and the finished products meet the storage capacity and safety stock limits in the industrial plants and the distribution hubs. Raw material *X*_2_ has a stock of 50 units because according to the bill of materials, its use is half that of raw material; therefore, in the production process, less units of *X*_2_ are consumed.

**Table 13:** Planned inventory level on supply chain echelons.

Month	Location	Item	Quantity
1	*I*_1_	*X*_1_	10
		*X*_2_	50
		*Y*_1_	10
		*Y*_2_	10
	*I*_2_	*X*_1_	10
		*X*_2_	50
		*Y*_1_	10
		*Y*_2_	10
	*H*_1_	*Y*_1_	10
		*Y*_2_	10
	*H*_2_	*Y*_1_	30
		*Y*_2_	20
2	*I*_1_	*X*_1_	10
		*X*_2_	50
		*Y*_1_	10
		*Y*_2_	10
	*I*_2_	*X*_1_	10
		*X*_2_	50
		*Y*_1_	10
		*Y*_2_	10
	*H*_1_	*Y*_1_	10
		*Y*_2_	10
	*H*_2_	*Y*_1_	10
		*Y*_2_	10

Table 12 presents the optimal transportation plan for each transport mode along the SC, considering the initial stocks, safety stocks, storage, and handling capacity of the SC echelons. Each transport mode can carry more than one type of product in each period. In this optimal plan, both the transport modes are used. Transport mode M1 is often engaged to its limit. Its capacity is half that of transport mode M2.

The optimal product delivery plan for each customer is listed in Table 10. However, information such as (i) the finished product flow from the distribution hubs and (ii) the quantity of products transported by each transport mode is complementary (Table 12). The inbound and outbound logistics in each hub are limited by the handling capacity of 50 units per month for each hub. Table 14 presents the dynamic use of handling resources.

**Table 14:** Input and output amount on hubs.

**Month**	**Hub**	**Quantity**	**Utilization**
1	*H*_1_	40	80%
	*H*_2_	50	100%
2	*H*_1_	10	20%
Input amount on distribution hubs			
**Month**	**Hub**	**Quantity**	**Utilization**
1	*H*_1_	30	60%
	*H*_2_	10	20%
2	*H*_1_	10	20%
	*H*_2_	30	60%
Output amount on distribution hubs			

Table 15 presents the optimal use of raw materials in an industrial plant and the quantity of the finished product obtained. Recalling the bill of materials of Fig 3, in plant *I*_1_, the production of 10 units of *Y*_1_ uses 20 units of *X*_1_ and 10 units of *X*_2_, whereas the production of 40 units of *Y*_2_ uses 80 units of *X*_1_ and 40 units of *X*_2_. Thus, the number of units of *X*_1_ used in plant *I*_1_ is 100 (20 + 80), whereas the number of units of *X*_2_ used in the same plant is 50 (10 + 40). In plant *I*_2_, the production of 50 units of *Y*_1_ uses 100 units of *X*_1_ and 50 units of *X*_2_.

**Table 15:** Raw materials consumption and production on plants.

**Month**	**Plant**	**Raw material**	**Quantity**
1	*I*_1_	*X*_1_	100
		*X*_2_	50
	*I*_2_	*X*_1_	100
		*X*_2_	50
Raw material consumption and production			
**Month**	**Plant**	**Product**	**Quantity**
1	*I*_1_	*Y*_1_	10
		*Y*_2_	40
	*I*_2_	*Y*_1_	50
Production on industrial plants			

Table 16 presents the production plan for each machine. To obtain the optimal solution, production is not activated in the second month. The high fixed costs of production contribute to the decision to force resource activation in the first month only. In addition, inventory accumulation in this period compensates for the resource deactivation in the second month. Each industrial plant makes 50 finished products, so, a total of 100 units are produced. Of these, 80 units are used to meet the demand and 20 units are allocated to the safety stock supply: 10 units for each plant (5 of *Y*_1_ and 5 of *Y*_2_).

**Table 16:** Production plan on industrial plants.

**Month**	**Plant**	**Resource**	**Activated?**	**Production**
1	*I*_1_	*M*_*A*_	Yes	50
		*M*_*B*_	Yes	50
	*I*_2_	*M*_*C*_	Yes	50
		*M*_*D*_	Yes	50
2	*I*_1_	*M*_*A*_	No	0
		*M*_*B*_	No	0
	*I*_2_	*M*_*C*_	No	0
		*M*_*D*_	No	0
Production plan by resource				
**Month**	**Plant**	**Resource**	**Product**	**Quantity**
1	*I*_1_	*M*_*A*_	*Y*_1_	10
		*M*_*A*_	*Y*_2_	40
		*M*_*B*_	*Y*_1_	10
		*M*_*B*_	*Y*_2_	40
	*I*_2_	*M*_*C*_	*Y*_1_	50
		*M*_*D*_	*Y*_1_	50
Detailed production plan by resource				

Although the procurement cost of *Y*_1_ is less than that of *Y*_2_ (Table 8), the variable production cost is lower in plant *I*_2_ (Table 7). Consequently, the entire available capacity in plant *I*_2_ is used to produce *Y*_1_. We realize that the activation costs of production resources are considerably higher than storage costs, so production is enabled only during the first month and is carried out at maximum capacity. Then, the finished products are transported to the distribution hubs where they remain in stock until the demand is met in the following month.

In comparison to traditional approaches, the proposed model present advantages on demonstrating quantitatively the impact of changes on each element on all echelon of SC, providing a holistic view to the managers responsible for SC tactical planning. The SC elements are, for example, the purchasing activity; the material used on production; the limited production or transportation capacity; or the demand in each period. For the robust-stochastic version, multiple scenarios can be simultaneously evaluated empowering decision-makers to select the tactical SC plan that best fits the global SC metrics, as presented in the results and discussion’s section.

This section proposed a numerical example to demonstrate the scope of the MESC-2SSP-RO formulation. The SC problem was based on two elements for each component for didactic purposes, however, solving the complete MILP problem with numerous elements is a challenge. The problem, namely dynamic lot-sizing, has been recognized to be NP-hard [31] since multiple product lots have to be planned within a capacity that varies over time.

## Results and discussion

The flexibility evaluation of the four-echelon SC is based on a three-dimensional framework, presented by Esmaeilikia et al. [28], and the tradeoff analysis of demand variability and service levels. The evaluation facilitates the investigation of the flexibility criteria for supply, manufacturing, and logistics. Supply flexibility includes make-or-buy and sourcing decisions. Manufacturing flexibility includes the production of multiple product types in each plant on machines and the expansion of production capacity. Logistics flexibility includes multimodal, transportation, handling, and storage strategies throughout the network. The literature on stochastic SC models that incorporate three-dimensional flexibility options is limited [28].

We considered a baseline stochastic example to evaluate three equiprobable scenarios [29] over 12 periods. The problem involves determining the optimal tactical plan for an SC with 3 suppliers, 2 industrial plants, 2 distribution hubs, and 10 customers. Four types of raw materials are processed in 5 machines in each plant, producing 10 types of finished products. Although this is a small problem, the stochastic four-echelon SC problem yields a big model; therefore, we did not describe the results in detail as we did in the previous deterministic numerical example. The model has 39,103 variables and 13,873 constraints with 9,652 continuous variables and 892 integer variables, of which 69 are binary. For evaluating flexibility by the comparison of scenarios, we present a report with only the aggregated values of financial and operational results (Table 17). Demand and price are the stochastic parameters. Demand is uniformly distributed with a minimum of 6 units and a maximum of 10 units, whereas the price is normally distributed with a mean of 100 units and a standard deviation of 10 units. The procurement cost of the raw material is $85.00/unit; 20 units are available with the suppliers. The fixed and variable costs for each plant are $500.00/(machine⋅period) and $20.00/unit, respectively. The overtime costs are $875.00/(machine⋅period), and the transportation costs are $2.50/(km⋅unit) for both the transport modes. For each period and plant, the safety stocks are set to 10 and 5 units for the raw materials and the finished products, respectively, and the safety stocks of finished products are set to 2 units in the distribution hubs. All the other parameters are kept constant in the following experiments.

**Table 17:** Performance of the baseline test-problem for flexibility evaluation.

Financial report	Value ($)	Operational report	Value (unit.)
Sales revenues	797,240.95	Raw material procurement	180,768
Logistics cost	190,400.00	Finished product procurement	0
Production fixed-cost	60,000.00	Inventory on supply chain	9,901
Production variable-cost	152,440.00	Production on plant-[1]	11,433
Procurement cost	30,128.00	Production on plant-[2]	11,433
Overtime cost	0.00	Transport on modal-[1]	172,746
Inventory cost	3,300.33	Transport on modal-[2]	55,734
Expected overall profit	360,972.62		
Profit scenario-[1]	361,959.75	Total demand	30,703
Profit scenario-[2]	374,603.85	Met demand	24,246
Profit scenario-[3]	346,354.25	Nonsatisfied demand	6,457

Under the above settings, the total demand is partially met. The industrial plants buy only raw materials from suppliers. Although 600 (20 × 10 × 3) finished products are available with suppliers in every period, the procurement cost of these products is not compensatory. The maximum capacity of manufacturing is used in the industrial plants, although extra capacity is not used. The items transported are assigned to both modal-[1] and modal-[2]. At the same costs, there is no preferable shipping mode between the two transport modes.

Stock-out situations may be affected by production lead times leading to non-satisfied demand. Therefore we conducted an additional experiment for the baseline test-problem changing the unit time required to produce product *p* on resource *r* on industrial plants, represented by the parameter $M_{lrp}^{C}$, to evaluate these joint effects. The reduction of up to 20% of production lead time reduced stock-out units form 6,457 to 3,718, while the increase in production lead time have unfavorable impacts on stock-out as presented in Table 18.

**Table 18:** Joint effect of stock-out and production lead time for baseline test-problem.

Leadtime	Delivery	Stock-out
-50%	26,985.00	3,718.00
-40%	26,985.00	3,718.00
-30%	26,985.00	3,718.00
-20%	26,985.00	3,718.00
-10%	26,476.60	4,226.40
**Base**	**24,246.00**	**6,457.00**
10%	22,076.30	8,626.70
20%	20,392.80	10,310.20
30%	18,870.20	11,832.80
40%	17,360.00	13,343.00
50%	16,042.00	14,661.00

Supply flexibility assesses strategies to choose suppliers based on the price of raw materials or their availability. It also evaluates the strategy to choose between making the finished product and buying it directly from a supplier. In this experiment, supply flexibility occurs when the company achieves a reduction in the procurement price of a finished product from $85.00/unit to $45.00/unit from one supplier. The results listed in Table 19 show that at this cost, the company can increase its profit by 16.74% by purchasing all the 600 finished products over 12 periods i.e. 7,200 units from suppliers, thereby increasing the overall service level and revenue. The costs of production and raw material procurement are reduced. Buying instead of producing finished products increases the overall profit by reducing both the consumption of manufacturing resources and the need to purchase raw materials.

**Table 19:** Performance of the test-problem with flexible supply.

Financial report	Value ($)	Operational report	Value (unit.)
Sales revenues	964,588.52	Raw material procurement	172,568
Logistics cost	199,858.33	Finished product procurement	7,200
Production fixed-cost	57,323.33	Inventory on supply chain	10,944
Production variable-cost	145,606.67	Production on plant-[1]	10,974
Procurement cost	136,761.33	Production on plant-[2]	10,867
Overtime cost	0.00	Transport on modal-[1]	186,799
Inventory cost	3,648.00	Transport on modal-[2]	53,031
Expected overall profit	421,390.85		
Profit scenario-[1]	418,951.60	Total demand	30,703
Profit scenario-[2]	442,929.84	Met demand	30,421
Profit scenario-[3]	402,291.12	Nonsatisfied demand	282

Manufacturing flexibility assesses volume flexibility and operational decisions. In this case, the tradeoffs between extra production costs and nonsatisfied demand costs are evaluated. We evaluate the manufacturing flexibility by reducing plant-[2]’s fixed costs of production by $100.00/(machine⋅period), reducing overtime costs by $250.00/(machine⋅period), and increasing the safety stock of raw materials from 10 units to 20 units in each industrial plant. Table 20 presents the financial and operational results. In this experiment, the average profit increases by 3.96% over the baseline scenario because of the reduction in operation costs and the increment on sales. The latter is attributed to the increase in production, which exceeds the available capacity. Thus, overtime is used mainly in plant-[2], which has a lower fixed cost of operation compared to plant-[1]. We also note that the variable cost of production increases and overtime is activated. On overtime activation, the consumption of raw materials increases. This change is operational, so there is no need to purchase finished goods from the supplier. The production in factories increases, so the quantity of inventory and goods transported throughout the chain also increases.

**Table 20:** Performance of the test-problem with flexible production volume.

Financial report	Value ($)	Operational report	Value (unit.)
Sales revenues	877,510.25	Raw material procurement	203,476
Logistics cost	214,004.17	Finished product procurement	0
Production fixed-cost	54,000.00	Inventory on supply chain	17,306
Production variable-cost	171,163.33	Production on plant-[1]	12,618
Procurement cost	33,912.67	Production on plant-[2]	13,056
Overtime cost	23,404.17	Transport on modal-[1]	178,953
Inventory cost	5,768.83	Transport on modal-[2]	77,852
Expected overall profit	375,257.08		
Profit scenario-[1]	376,319.75	Total demand	30,703
Profit scenario-[2]	392,915.10	Met demand	27,054
Profit scenario-[3]	356,536.40	Nonsatisfied demand	3,648

Logistics flexibility assesses the ability to adopt alternative strategies for transport and storage to meet the customer demand on time. In this experiment, the logistics flexibility is evaluated by reducing the distribution cost for modal-[2] from $2.50/(km⋅unit) to $2.40/(km⋅unit). Additionally, we change the inventory safety level of finished products from 2 units to 5 units in distribution centers. The financial and operational results are presented in Table 21. In previous experiments, products are allocated to a transport mode by considering only its capacity because there is no difference in the transportation costs. In this case, there is neither purchase of products nor overtime activation. Revenue, production costs, and purchases are not altered compared to the baseline scenario. Nevertheless, the transportation of units is concentrated on modal-[2] because of its lower cost. The increase in the total expected profit is a consequence of the reduced distribution costs. However, a part of this profit is consumed by the cost of maintaining higher safety stocks of finished products in distribution centers.

**Table 21:** Performance of the test-problem with flexible logistics.

Financial report	Value ($)	Operational report	Value (unit.)
Sales revenues	792,132.80	Raw material procurement	180,768
Logistics cost	184,345.60	Finished product procurement	0
Production fixed-cost	60,000.00	Inventory on supply chain	11,727
Production variable-cost	152,440.00	Production on plant-[1]	11,433
Procurement cost	30,128.00	Production on plant-[2]	11,433
Overtime cost	0.00	Transport on modal-[1]	51,168
Inventory cost	3,909.00	Transport on modal-[2]	177,132
Expected overall profit	361,310.20		
Profit scenario-[1]	362,284.15	Total demand	30,703
Profit scenario-[2]	374,685.80	Met demand	24,066
Profit scenario-[3]	346,960.65	Nonsatisfied demand	6,637

**Fig 4:**
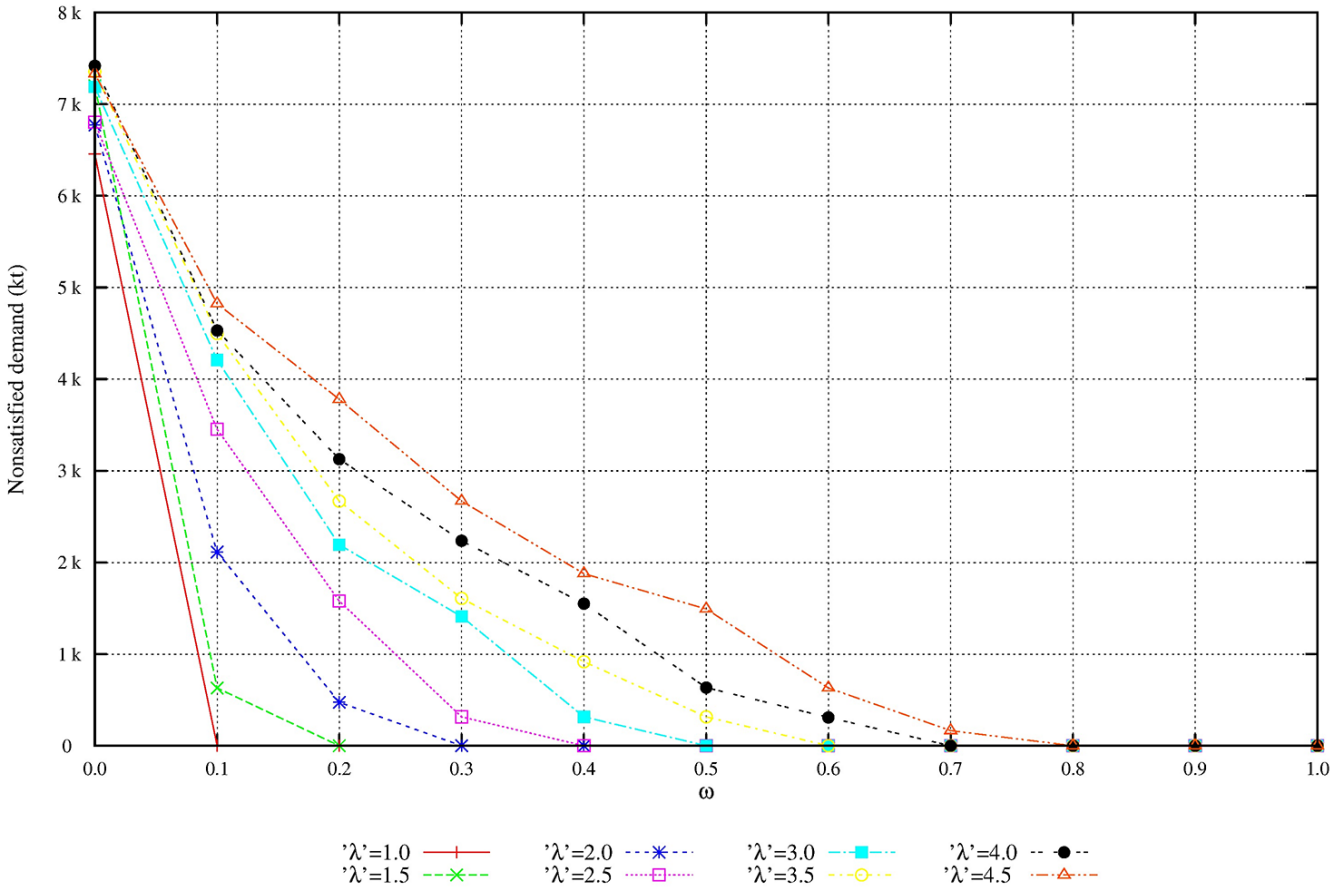
Service level with the increase of penalty *ω*. Nonsatisfied demand reduces due to the increase of penalty *ω*: Model robustness.

**Fig 5:**
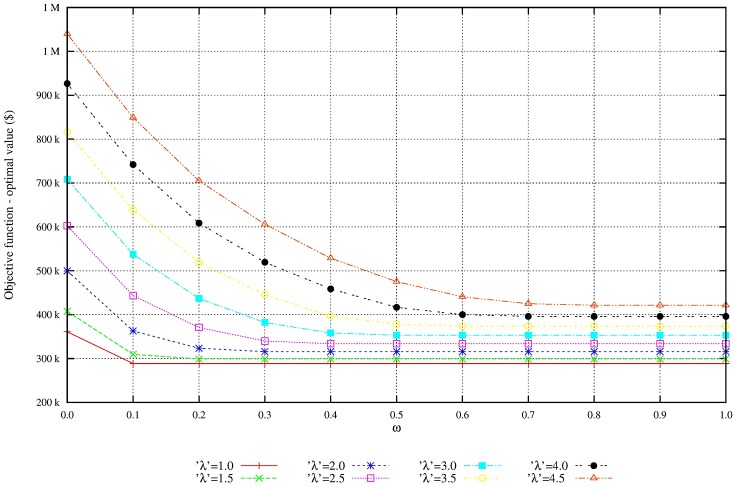
Overall profit by increasing of penalty *ω*. Monotonic reduction of the total profit due to the increase of penalty *ω*: Solution robustness.

**Fig 6:**
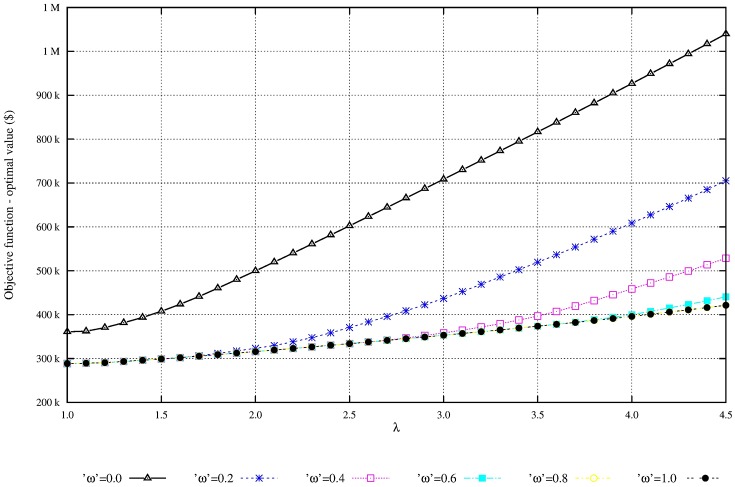
Overall profit of scenarios with variability λ. Increase in total profit due to the increase on demand variability λ): Solution robustness.

**Fig 7:**
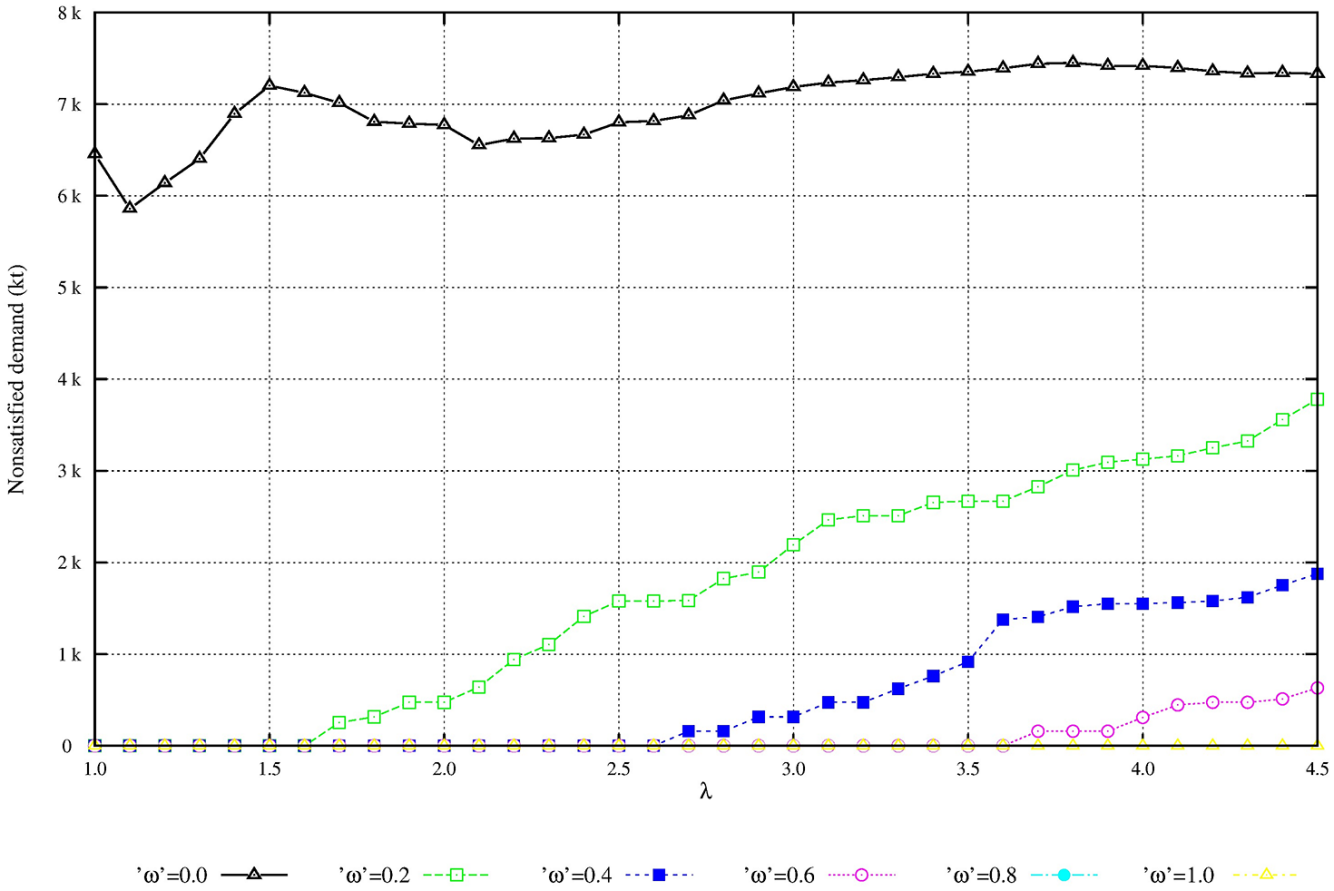
Service level of scenarios with variability λ. Increase of nonsatisfied demand due to the increase of variability λ): Model robustness.

We perform additional experiments to evaluate the solution robustness and the model robustness. The quality of flexibility and tradeoff analysis is enhanced by setting the parameters of demand variability, λ, and the penalty for nonsatisfied demand, *ω*. Figs 4 and 5 shows that as the penalty for nonsatisfied demand increases, the total expected profit is reduced and the unmet demand is also reduced. Figs 6 and 7 shows that under great demand variability, the nonsatisfied demand increases; however, the global expected profit of the SC can be enhanced by selecting profitable orders and increasing the inventory level over SC echelons. Such a strategy increases the availability of products to customers. The rise in the inventory costs over SC echelons is compensated by more sales.

Decision makers aim to evaluate supply, manufacturing, and distribution scenarios and select the option that provides the best contribution to the global SC metrics. For managers, it is important to know the quantity of materials and products that should be bought, produced, stocked, transported, and sold over different periods. The results show that demand variability and the service level are in conflict with each other. Our proposed robust stochastic programming model helps decision makers to evaluate supply, manufacturing, and distribution strategies, decide as to which service level should be selected from the perspective of demand variability, and understand the impact of a decision on the overall expected profit. Moreover, our proposed tactical model provides strategic decisions in case of SC disruptions. Strikes, accidents, or supply shortage can occur; in such cases, managers can, for example, plan optimal changes in the transport mode, material flow on machines, or supplier used.

## Conclusion

This study developed a robust stochastic programming model to evaluate the flexibility of a four-echelon SC at a tactical level. The proposed model simultaneously considered demand variability and service levels for scenarios of flexibility in supply, production, and distribution. Although such a strategy increases the model size and its complexity, the approach fills the gaps in the literature pertaining to the flexibility analysis of an integrated SC and is aligned to current research trends.

The supply flexibility evaluated the strategy to buy the finished product directly from a supplier. In this case, the company acts as a link, activating only its logistics infrastructure to meet demand. This situation occurs when the company’s fixed costs of operational activation do not compensate for the level of demand. However, the company runs the risk of having its image damaged by providing a competitor’s product. The manufacturing flexibility evaluated the strategy of reducing fixed costs of machines and overtime costs in plant-[1]. From this analysis, the idea of increasing production in plant-[2] emerged because the product families can be manufactured in both the industrial plants to prevent stock disruption. The logistics flexibility evaluated a reduction in the distribution cost for modal-[2] and an increase in the inventory safety level. The reduction in the distribution costs increased the total expected profit, but a part of it was consumed by the cost of maintaining higher safety stocks in distribution centers. Finally, additional experiments were performed to assess the flexibility and the robustness simultaneously. The proposed approach facilitates the adoption of different management styles and improves SC resilience. The model can be extended to contexts pertaining to SC disruptions by exploring strategies when unexpected events disrupt the supply, manufacturing, or distribution network of a company.

In summary, the main features of this paper are as follows: (1) introduction of a robust stochastic programming formulation to a four-echelon SC model; (2) consideration of parameter uncertainty for the evaluation of flexibility in three dimensions: supply, production, and distribution; and (3) increase in SC resilience based on flexibility analysis that considers the tradeoff between demand variability and the service level.

Although we have demonstrated the advantages of our model, our study has some limitations. Backlogging is not considered, and each product has one technical route. In future research, the stochastic parameters of the transportation lead time and production can be considered. The study of a risk-averse robust formulation is also a future research avenue. Moreover, as the amount of data grows, this problem, which is nondeterministic polynomial-time hard, becomes difficult to solve. Future developments can include the development of an efficient method of decomposition for solving the large-scale multiechelon SC planning problem.

## References

[1] Gunasekaran A, Subramanian N, Rahman S. Supply chain resilience: role of complexities and strategies. International Journal of Production Research. 2015;53(22):6809–6819. doi: 10.1080/00207543.2015.1093667

[2] Wallace SW. Decision making under uncertainty: Is sensitivity analysis of any use? Operations Research. 2000;48(1):20–25. doi: 10.1287/opre.48.1.20.12441

[3] Higle JL. Stochastic programming: optimization when uncertainty matters. Cole Smith J (ed) Tutorials in operations research. 2005; p. 30–53.

[4] De Neufville R, Scholtes S. Flexibility in engineering design. MIT Press; 2011.

[5] Dantzig GB. Linear programming under uncertainty. Management science. 1955;1(3-4):197–206. doi: 10.1287/mnsc.1.3-4.197

[6] Birge JR, Louveaux F. Introduction to stochastic programming. Springer Science & Business Media; 2011.

[7] King AJ, Wallace SW. Modeling with stochastic programming. Springer Science & Business Media; 2012.

[8] Birge JR. State-of-the-art-survey-stochastic programming: Computation and applications. INFORMS journal on computing. 1997;9(2):111–133. doi: 10.1287/ijoc.9.2.111

[9] Mula J, Poler R, Garcia-Sabater J, Lario F. Models for production planning under uncertainty: A review. International journal of production economics. 2006;103(1):271–285. doi: 10.1016/j.ijpe.2005.09.001

[10] Sodhi MS, Tang CS. Modeling supply-chain planning under demand uncertainty using stochastic programming: A survey motivated by asset–liability management. International Journal of Production Economics. 2009;121(2):728–738. doi: 10.1016/j.ijpe.2009.02.009

[11] Soyster AL. Technical note—convex programming with set-inclusive constraints and applications to inexact linear programming. Operations research. 1973;21(5):1154–1157. doi: 10.1287/opre.21.5.1154

[12] Falk JE. Technical Note—Exact Solutions of Inexact Linear Programs. Operations Research. 1976;24(4):783–787. doi: 10.1287/opre.24.4.783

[13] Gabrel V, Murat C, Thiele A. Recent advances in robust optimization: An overview. European journal of operational research. 2014;235(3):471–483. doi: 10.1016/j.ejor.2013.09.036

[14] Mulvey JM, Vanderbei RJ, Zenios SA. Robust optimization of large-scale systems. Operations research. 1995;43(2):264–281. doi: 10.1287/opre.43.2.264

[15] Ben-Tal A, Nemirovski A. Robust convex optimization. Mathematics of Operations Research. 1998;23(4):769–805. doi: 10.1287/moor.23.4.769

[16] Sahinidis NV. Optimization under uncertainty: state-of-the-art and opportunities. Computers & Chemical Engineering. 2004;28(6):971–983. doi: 10.1016/j.compchemeng.2003.09.017

[17] Alem D, Morabito R. Risk-averse two-stage stochastic programs in furniture plants. OR spectrum. 2013;35(4):773–806. doi: 10.1007/s00291-012-0312-5

[18] Gupta A, Maranas CD. Managing demand uncertainty in supply chain planning. Computers & Chemical Engineering. 2003;27(8):1219–1227. doi: 10.1016/S0098-1354(03)00048-6

[19] Yu CS, Li HL. A robust optimization model for stochastic logistic problems. International Journal of Production Economics. 2000;64(1):385–397. doi: 10.1016/S0925-5273(99)00074-2

[20] Ben-Tal A, Golany B, Nemirovski A, Vial JP. Retailer-supplier flexible commitments contracts: A robust optimization approach. Manufacturing & Service Operations Management. 2005;7(3):248–271. doi: 10.1287/msom.1050.0081

[21] Pishvaee MS, Rabbani M, Torabi SA. A robust optimization approach to closed-loop supply chain network design under uncertainty. Applied Mathematical Modelling. 2011;35(2):637–649. doi: 10.1016/j.apm.2010.07.013

[22] Lalmazloumian M, Wong KY, Govindan K, Kannan D. A robust optimization model for agile and build-to-order supply chain planning under uncertainties. Annals of Operations Research. 2013; p. 1–36.

[23] Kristianto Y, Gunasekaran A, Helo P, Hao Y. A model of resilient supply chain network design: A two-stage programming with fuzzy shortest path. Expert Systems with Applications. 2014;41(1):39–49. doi: 10.1016/j.eswa.2013.07.009

[24] Chiu SW, Chen SW, Chang CK, Chiu YSP. Optimization of a Multi–Product Intra-Supply Chain System with Failure in Rework. PloS one. 2016;11(12):e0167511 doi: 10.1371/journal.pone.0167511 27918588 10.1371/journal.pone.0167511 PMC5137895

[25] Avci MG, Selim H. A Multi-objective, simulation-based optimization framework for supply chains with premium freights. Expert Systems with Applications. 2017;67:95–106. doi: 10.1016/j.eswa.2016.09.034

[26] Amin SH, Zhang G, Akhtar P. Effects of uncertainty on a tire closed-loop supply chain network. Expert Systems with Applications. 2017;73:82–91. doi: 10.1016/j.eswa.2016.12.024

[27] Pedram A, Pedram P, Yusoff NB, Sorooshian S. Development of closed–loop supply chain network in terms of corporate social responsibility. PloS one. 2017;12(4):e0174951 doi: 10.1371/journal.pone.0174951 28384250 10.1371/journal.pone.0174951 PMC5383151

[28] Esmaeilikia M, Fahimnia B, Sarkis J, Govindan K, Kumar A, Mo J. Tactical supply chain planning models with inherent flexibility: definition and review. Annals of Operations Research. 2016;244(2):407–427. doi: 10.1007/s10479-013-1513-2

[29] Dupačová J, Consigli G, Wallace SW. Scenarios for multistage stochastic programs. Annals of operations research. 2000;100(1-4):25–53.

[30] Pochet Y, Wolsey LA. Production planning by mixed integer programming. Springer; 2006.

[31] Bitran, Gabriel R and Yanasse, Horacio H. Computational complexity of the capacitated lot size problem Management Science 1982; 28(10): 1174–1186. doi: 10.1287/mnsc.28.10.1174

