# Correction: Flexibility evaluation of multiechelon supply chains

**DOI:** 10.1371/journal.pone.0198718

**Published:** 2018-06-01

**Authors:** João Flávio de Freitas Almeida, Samuel Vieira Conceição, Luiz Ricardo Pinto, Ricardo Saraiva de Camargo, Gilberto de Miranda Júnior

There are errors in affiliations 1 and 2 for the authors. For João Flávio de Freitas Almeida, Samuel Vieira Conceição, Luiz Ricardo Pinto, and Ricardo Saraiva de Camargo, affiliation 1 should be: Dept. of Industrial Engineering, Universidade Federal de Minas Gerais, Belo Horizonte, Minas Gerais, Brazil. For Gilberto de Miranda Júnior, affiliation 2 should be: Dept. of Applied Mathematics, Universidade Federal do Espírito Santo, São Mateus, Espírito Santo, Brazil.

## References

[1] Almeida JFdF, Conceição SV, Pinto LR, de Camargo RS, Júnior GdM (2018) Flexibility evaluation of multiechelon supply chains. PLoS ONE 13(3): e0194050 https://doi.org/10.1371/journal.pone.0194050 29584755 10.1371/journal.pone.0194050 PMC5871325

